# Machine Learning Approach to Vertical Energy Gap in Redox Processes

**DOI:** 10.1021/acs.jctc.4c00715

**Published:** 2024-07-24

**Authors:** Ronit Sarangi, Suman Maity, Atanu Acharya

**Affiliations:** † Department of Chemistry, Syracuse University, Syracuse, New York 13244, United States; ‡ BioInspired Syracuse, Syracuse University, Syracuse, New York 13244, United States

## Abstract

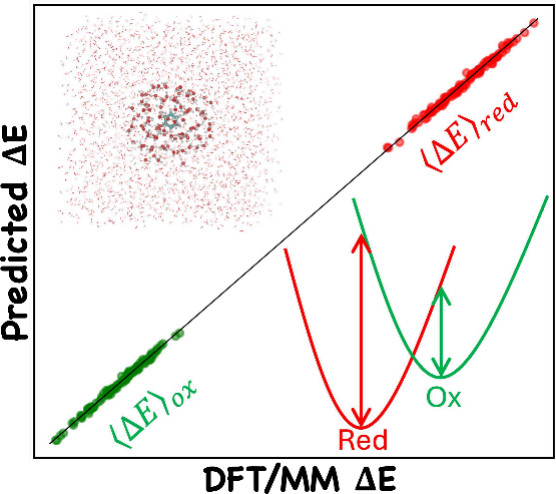

A straightforward approach to calculating the free energy change (Δ*G*) and reorganization energy of a redox process is linear response approximation (LRA). However, accurate prediction of redox properties is still challenging due to difficulties in conformational sampling and vertical energy-gap sampling. Expensive hybrid quantum mechanical/molecular mechanical (QM/MM) calculations are typically employed in sampling energy gaps using conformations from simulations. To alleviate the computational cost associated with the expensive QM method in the QM/MM calculation, we propose machine learning (ML) methods to predict the vertical energy gaps (VEGs). We tested several ML models to predict the VEGs and observed that simple models like linear regression show excellent performance (mean absolute error ∼0.1 eV) in predicting VEGs in all test systems, even when using features extracted from cheaper semiempirical methods. Our best ML model (extra trees regressor) shows a mean absolute error of around 0.1 eV while using features from the cheapest QM method. We anticipate our approach can be generalized to larger macromolecular systems with more complex redox centers.

## Introduction

Redox free energy defines the energy required to add or remove an electron from a molecule in a solution. It provides vital insights into a myriad of chemical and biological processes, including spontaneity and direction of electron transfer reactions, electrochemical catalysis, design of redox flow batteries,^1,2^ and mechanism of biological electron transfer.^3−6^ Traditionally, the calculation of redox free energy, Δ*G*_*r*_, has relied on methods such as the free energy perturbation (FEP)^7^ or thermodynamic integration (TI).^8,9^ Both techniques involve extensive sampling along the intermediate states that connect the reduced and oxidized states. A large number of intermediate states are required to ensure overlap between states in FEP.^10,11^

The linear response approximation (LRA)^12,13^ presents an alternative approach, reducing the computational burden by focusing on only the two end states–the reduced (red) and oxidized (ox) states, and the equation for Δ*G*_*r*_ becomes,
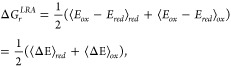
1where ΔE is the vertical energy gap (VEG) between the oxidized species and the reduced species, and ⟨···⟩_*red*_ implies that the VEG is averaged over the reduced surface. However, we need to use high-level quantum chemical calculations for thousands of conformations to obtain a reliable estimate of a single ensemble-averaged ΔE value.

Other challenges stem from the need to account for the open-shell character of oxidized/reduced species and to incorporate environmental effects^14^ into the VEG calculation. The former issue is addressed using high-level quantum chemical methods reported to handle closed and open-shell species on an equal footing. The latter issue can be resolved by using embedding methods such as electrostatic embedding,^15^ effective fragment potentials,^16−18^ or a hybrid QM/MM calculation with a large QM region.^19^ Thus, using quantum chemical calculations to estimate VEGs, and in turn, the redox potentials, is a challenging and computationally expensive task.^20,21^

In recent years, the application of machine learning (ML) techniques in chemistry has revolutionized various aspects of computational chemistry and emerged as an alternative to expensive quantum chemical calculations^22,23^ for energy^24,25^ and dynamics.^26−28^ ML models, such as kernel ridge regression (KRR), random forests (RF), and neural networks (NN), have shown remarkable success in predicting redox potentials,^29,30^ ionization energies,^31^ electron affinities,^32^ and coupled-cluster singles and doubles with perturbative triples (CCSD(T)) energies with high accuracy and speed.^33,34^ These models leverage large data sets of molecular structures to learn the correlation between structure and properties. This paradigm shift offers a potential solution to the challenges posed by traditional computational chemistry methods, providing a more efficient and scalable approach for predicting redox properties for various chemical systems, as demonstrated by several studies.^29,35,36^ For a comprehensive overview, refer to a recent review by Fedorov and Grynóva.^30^

**Figure 1:**
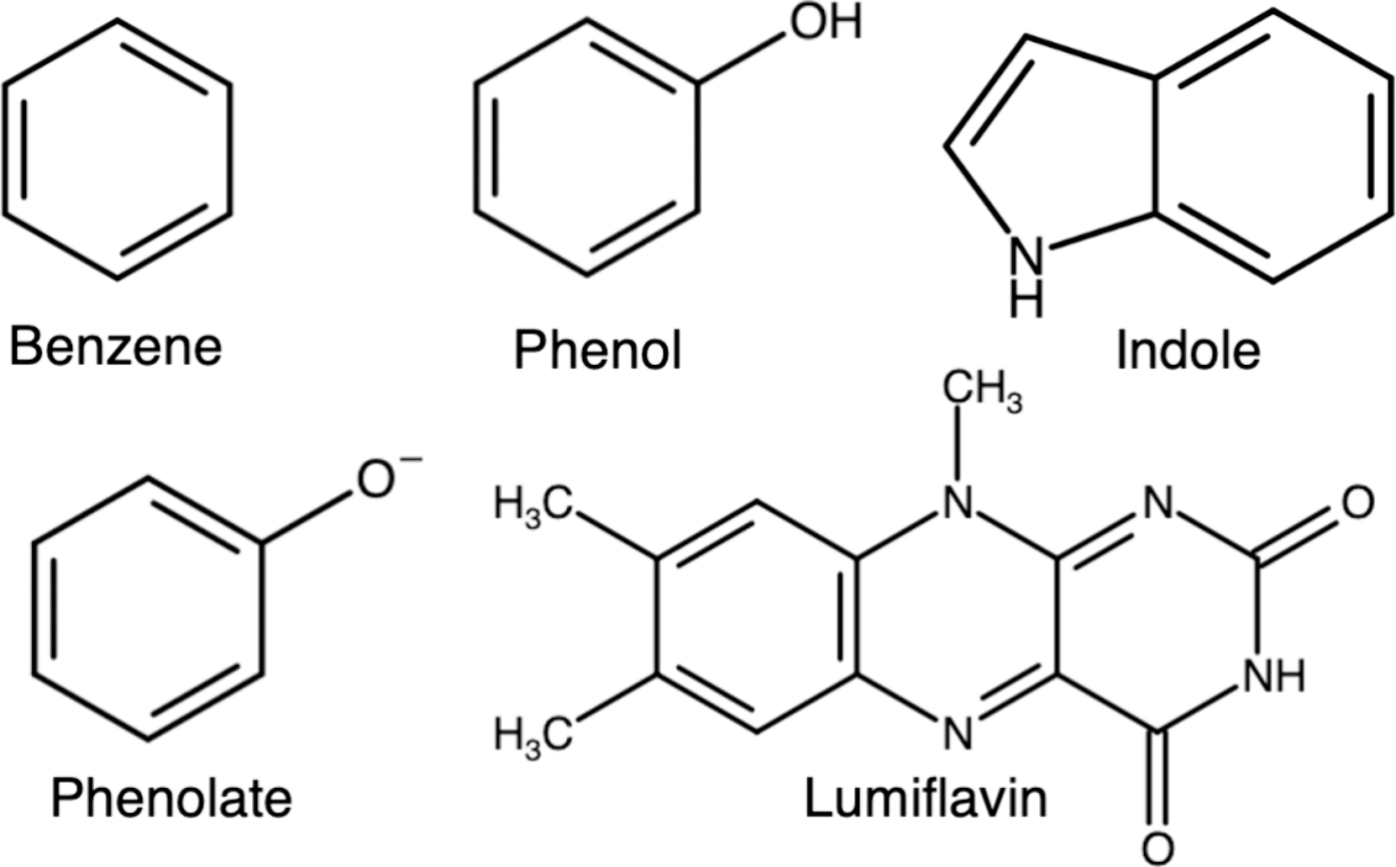
Model systems used in this study.

In this work, we adopt a different approach and develop ML models to predict VEGs for a set of small biologically relevant molecules at different geometries of the solute and solvent. We consider 5 systems in an aqueous environment: benzene, phenol, phenolate ion, indole, and lumiflavin. Figure 1 shows the reduced state of the systems used in this study, except for lumiflavin, which is shown in its oxidized state. The redox properties of species like phenol and phenolate are important since they act as redox-active center of the amino acid tyrosine. Similarly, indole and benzene are part of the amino acids tryptophan and phenylalanine, respectively. Lumiflavin belongs to a family of flavins,^37−39^ which function as a redox center in many biological processes.^40,41^ Herein, we used features extracted from inexpensive quantum chemical calculations to predict VEGs, which can then be used for redox free energy calculation according to eq 1.

## Methods

### Computational Details

Initial force field parameters were obtained from CGenFF for the reduced (oxidized for lumiflavin) states, followed by necessary modifications. The force field design is discussed in detail in the Supporting Information (SI). We built a periodic cubic box of size 50 × 50 × 50 Å^3^ for the QM/MM simulations, with the solute at the center and a counterion, if necessary, to make the full system charge neutral. The box size was selected to account for the long-range solvation effects on VEGs, as reported in ref (15). The solvent density was equilibrated in a 2 ns constrained MD simulation with 1 fs time step, where the solute molecule remains fixed. The equilibrated system was divided into QM and MM regions, with the QM region containing the solute of interest and the MM region containing TIP3P waters and any counterions. The QM region was described by the BP86/6-31+G* level of theory, and the simulation was run using the NAMD-ORCA^42−44^ interface. The system was equilibrated for 5 ps, where everything was allowed to relax, followed by ten replicates of 5 ps QM/MM simulation (total 50 ps) in an NPT ensemble with 0.5 fs time step. For all simulations, the temperature and pressure were kept at 310 K and 1 atm, respectively. For each system, 500 snapshots were extracted from the 50 ps QM/MM simulations for single-point energy calculations and ML training.

### Single-Point Energy Calculations

The snapshots collected from the QM/MM sampling of reduced and oxidized states were used to calculate VEGs using a QM/MM approach with an electrostatic embedding scheme. The energy gaps were computed at three levels of theory–wB97MV/6-31+G*,^45^ HF/6-31+G*, and HF-3c/MINIX^46^ for the QM region and the MM region was included as point charges. We refer to the HF-3c/MINIX level of theory as semiempirical (EMP) for the rest of the article. We used the HF-3c as our semiempirical method of choice since it accounts for dispersion correction (D3).^47^ Furthermore, HF-3c method is shown to be numerically stable for a wide range of systems including biomolecules.^46,48,49^ The wB97MV functional is also a dispersion-corrected meta-GGA functional and presents the lowest error (∼0.11 eV) in ionization energy for a large set of molecules.^45^ Therefore, the VEGs extracted from the density functional theory (DFT) calculations were considered the reference, and the ML features were extracted from the relatively cheaper HF and semiempirical (EMP) calculations. We also performed equation-of-motion coupled-cluster with singles and doubles excitations for ionization potential (known as EOM-IP-CCSD)^50^ calculation on a small subset of our systems to demonstrate that features calculated using HF/MM or EMP/MM can be used to predict VEGs at post-HF level. Due to the high computational cost, EOM-IP-CCSD calculations were performed only on the reduced surface of benzene, phenol, phenolate, and indole using a QM cutoff of 0.0 Å.

The dependence of VEGs on the size of the QM system was explored in terms of two cutoff radii for the construction of the QM region. Only the solute was included in the QM region for the cutoff radius of 0 Å (“QM cutoff 0.0”). In contrast, for cutoff radius 7.5 Å (“QM cutoff 7.5”), we included the solute and all waters within 7.5 Å of the center of mass of the solute. The average number of water molecules included in the QM region is listed in Table S1.

The VEGs obtained from the DFT/MM and HF/MM single point energy calculations are collected in Table 1, and the EMP/MM VEGs are shown in the SI. All single-point QM/MM calculations were carried out using the Q-Chem^51^ package.

**Table 1:** VEGs (in eV) of all systems calculated using HF/MM and ωB97MV/MM for “QM cutoff 0.0” and “QM cutoff 7.5”. The 6-31+G* basis set was used for both QM methods

		QM cutoff 0.0		QM cutoff 7.5	
Molecule	Surface	HF/MM	ωB97MV/MM	HF/MM	ωB97MV/MM
Benzene	Reduced	8.09 ± 0.35	9.44 ± 0.34	7.66 ± 0.35	9.03 ± 0.34
	Oxidized	3.34 ± 0.35	4.72 ± 0.34	3.17 ± 0.34	4.76 ± 0.32
Phenol	Reduced	7.73 ± 0.38	8.89 ± 0.37	7.24 ± 0.38	8.45 ± 0.36
	Oxidized	2.69 ± 0.33	3.99 ± 0.32	2.43 ± 0.33	3.92 ± 0.30
Phenolate	Reduced	6.10 ± 0.39	7.59 ± 0.37	6.28 ± 0.38	7.81 ± 0.34
	Oxidized	0.91 ± 0.36	2.57 ± 0.34	1.30 ± 0.38	3.11 ± 0.34
Indole	Reduced	6.79 ± 0.39	8.16 ± 0.36	6.35 ± 0.38	7.79 ± 0.35
	Oxidized	2.14 ± 0.38	3.64 ± 0.35	1.95 ± 0.38	3.67 ± 0.32
Lumiflavin	Reduced	6.29 ± 0.40	6.12 ± 0.35	6.35 ± 0.40	6.24 ± 0.35
	Oxidized	2.27 ± 0.38	2.31 ± 0.34	2.42 ± 0.38	2.58 ± 0.34

### Machine Learning Models

ML models were used to predict the VEGs calculated from DFT/MM with features extracted using cheaper calculations such as HF/MM or EMP/MM methods. Specifically, the reference for the models was the energy gap calculated using the wB97MV/6-31+G* method at QM cutoff radius 7.5 Å, and the features were extracted from HF/6-31+G* and HF-3c/MINIX method at QM cutoff radius 0.0 Å. Each system had 1000 data points (500 snapshots for each state) that the ML model could train on, and because of the small data set size, we chose eight physics-inspired features for training: Nuclear repulsion energy (NRE) and dipole moment of the solute capture the effects of solute conformations. The highest occupied molecular orbital (HOMO) and the lowest unoccupied molecular orbital(LUMO) energies capture the effects of orbital energies on VEGs. We selected three features to capture the solute–solvent interactions, the charge–charge electrostatic interaction energy between atoms in the MM region, total electrostatic potentials^52^ at 5 Å (esp5) and 10 Å (esp10) from the solute. Finally, the total HF/semiempirical SCF energy was selected as the last feature, following the ΔML approach introduced by Ramakrishnan et al.^53^

**Figure 2:**
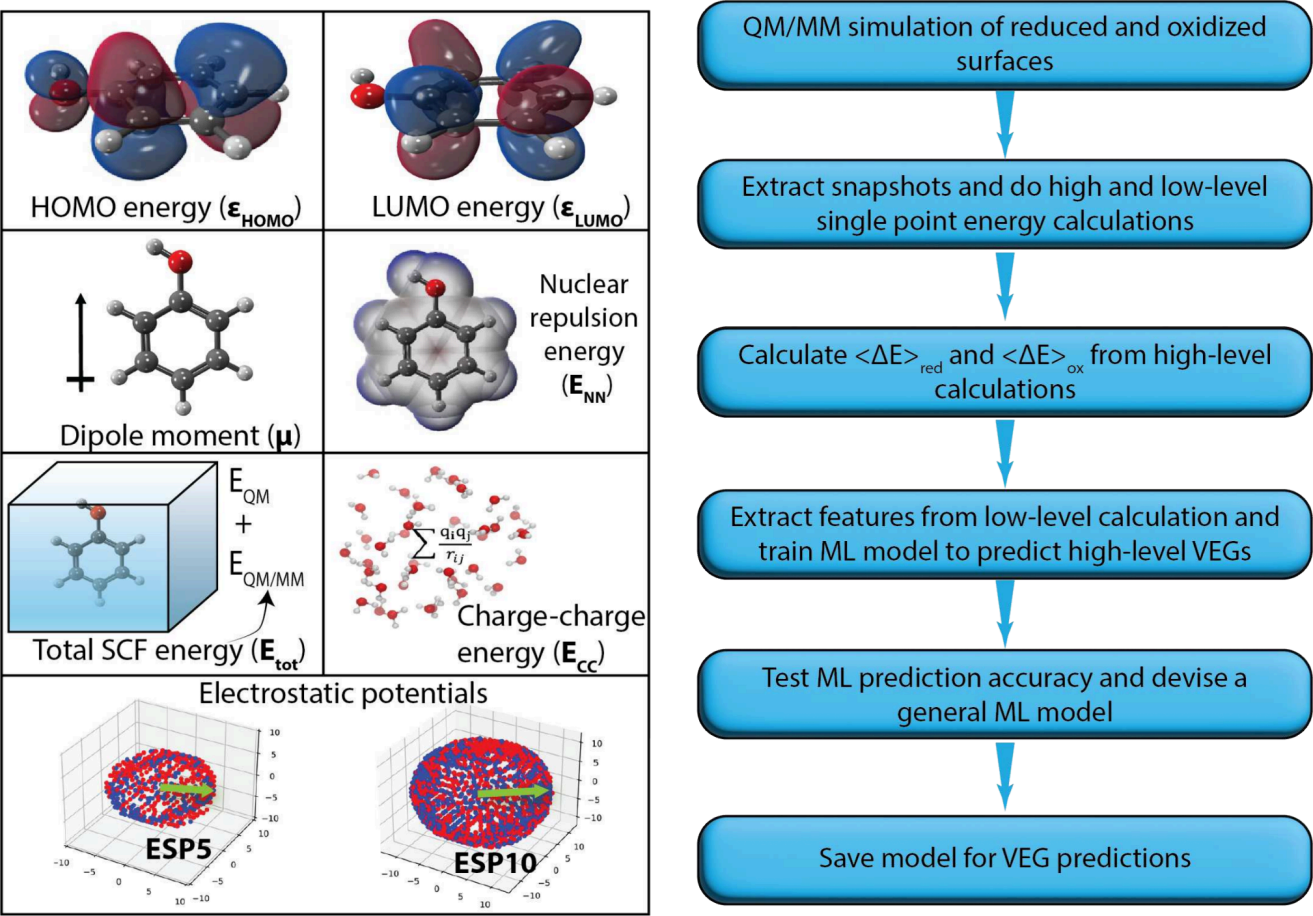
ML features used for training (left) and the pipeline used in this study to generate data, train, and test ML models (right).

The data set was split randomly into 80% train and 20% test sets and scaled to zero mean and unit variance over the training set. Two sets of models were designed: the first set consisted of models for individual systems of interest, i.e., five in total, and the second set consisted of a general model that could be used to predict the ΔE values of all systems in this study. Additional details about the features are provided in the SI. We used the scikit-learn^54^ package to train five different ML models: linear regression (LR), polynomial regression (PR, degree 3), kernel ridge regression (KRR), multilayer perceptron (MLP), and extra trees regressor (ETR)^55^ for both sets. Except for linear regression models, the hyperparameter optimization was carried out for the rest using a nested 5-fold cross validation using the *GridSearchCV* function in scikit-learn. The performance of the models with optimized hyperparameters was evaluated using 5-fold cross validation using the *cross_validate* function in scikit-learn. After evaluating all the models, they were trained and tested using 80/20 split and saved for further testing. The pipeline used in this study, along with the features, is shown in Figure 2.

## Results and Discussions

In this section, we will discuss the trends in the VEGs for different QM methods and the selection of QM regions. We will also compare our results with experimental reference, where available. We will then analyze ML models in terms of accuracy scores such as mean absolute error (MAE) and root-mean-square error (RMSE) in the VEGs. Using learning curves, we also investigate the number of snapshots required to build an ML model that can predict VEGs with a reasonable accuracy score. We will then compare the models trained using features extracted from HF/MM and EMP/MM methods. Finally, we explore the behavior of simple models like linear regression for individual systems compared to their performance for “All” systems.

### VEG (ΔE) Calculations

Table 1 shows the comparison between HF/MM and ωB97MV/MM ΔEs calculated at the QM cutoff values of 0.0 Å (only the solute in the QM region) and 7.5 Å (solute + all waters within 7.5 Å from the center of mass of the solute in the QM region). The respective VEGs for EMP/MM calculations are shown in the SI (Table S4). The comparison reveals that HF/MM generally underestimates VEG values compared to DFT/MM, except for the reduced state of lumiflavin. Additionally, we observe that the variance of VEGs remains relatively stable across both methods, as shown in the distribution of VEG values (Figure S1). Typically, DFT describes open-shell doublet species better, exhibiting reduced spin-contamination compared to HF and EMP calculations.^56,57^ Analysis of ⟨*S*^2^⟩ values (Table S5) shows the DFT/MM calculations are closer to the ideal ⟨*S*^2^⟩ value for doublets (0.75) than either HF/MM or EMP/MM methods. Additionally, the spread in the ⟨*S*^2^⟩ values is minimal in DFT/MM, whereas there is a large range of ⟨*S*^2^⟩ values for the other calculations.

We observed that the benzene, phenol, and indole VEG values calculated on the reduced surface (closed shell, neutral) shifts to lower energy by about 0.3–0.4 eV on going from cutoff 0.0 to 7.5. The same value calculated on the oxidized surface (open shell, cation) displays a slight shift of approximately 0.03–0.04 eV. This trend is reversed in phenolate and lumiflavin, both of which have neutral oxidized states and exhibit a shift toward higher energy of about 0.2–0.5 eV on going to a larger QM region, whereas their reduced state (charged) shows a smaller shift. This indicates that neutral species are more susceptible to alterations in cutoff radius than their charged counterparts. A similar observation was reported in a study by Tazhigulov and Bravaya,^15^ where they observed a linear relationship between VEGs and inverse radius of the solvation sphere. Furthermore, we observed that a small variation of the number of water molecules between frames does not affect the VEGs significantly (Table S2). The small changes between frames originate from the different solute and solvent geometries. To delineate the effect of the solvent, we selected one frame for each molecule and calculated VEGs with ±2 water molecules relative to the 7.5 Å QM cutoff. For the same frame, VEG values are insensitive to small variations in the number of water, as shown in Table S3. Although these observations provide valuable insight into the behavior of molecular systems under different computational protocols, investigating deeper into the precise mechanism exceeds the scope of this study.

Among the three types of calculations (HF/MM, DFT/MM, and EMP/MM), the DFT/MM calculations at a QM cutoff 7.5 Å is chosen as the reference for ML prediction in this study because, in our case, DFT presents less spin-contaminated results and the higher QM region cutoff includes the effects of environment explicitly. The larger QM region also accounts for solute–solvent polarization explicitly. A similar approach was used in the study by Sterling and Bjornsson,^21^ where they found a QM region with ∼50 waters for small organic molecules (includes solvent within 5 Å from any solute atom) was enough to account for solvent polarization.

### Computational vs Experimental Ionization Energies

Table 2 shows the comparison between the computed and experimental vertical ionization energies (VIE). We use the VEG calculated on the reduced surface at ωB97MV/MM level of theory with a QM cutoff of 7.5 Å as the VIE. The computed VEG value for benzene aligns closely with the experimental VIE value for a microsolvated benzene with one water molecule.

**Table 2:** Comparison of computational vertical energy gap (VEG) and experimental vertical ionization energies. VEG is defined as  for this comparison

Molecule	Comp. VEG (eV)	Expt. VIE (eV)
Benzene	9.03	9.170 ± 0.014^a^^,^^58^
		9.186 ± 0.007^a^^,^^59^
Phenol	8.45	∼7.3–8.3^16^
Phenolate	7.81	7.1 ± 0.1^16^
Indole	7.79	7.4 ± 0.1^60^

a Benzene-1 water cluster.

The experimental VIE estimate of phenol is quite ambiguous because of an extremely high overlap between the first and second ionization (7.8 to ∼8.5 eV) in the valence photoelectron spectra of phenol (Figure 9 in ref (16)). The first ionization energy has a peak at 7.8 ± 0.1 eV, for phenol, with ∼1 eV full width at half-maximum. Therefore, we take the experimental VEG range as 7.3 to 8.3 eV in our comparisons. The same article also reports the valence photoelectron spectra of aqueous phenolate. The first ionization peak is much more distinctive at 7.1 eV. The VEG of phenolate was previously estimated to be 7.7 eV at EOM-CCSD level of theory with effective water fragment potential (EFP) treatment of water.^16^ Therefore, the EOM-CCSD VEG estimate needed to be shifted by 0.8 eV to match the experimental spectrum even with the high-level theory and polarizable environment. In this work, the computed VEG of phenol and phenolate at ωB97MV/MM are 8.45 and 7.81 eV, respectively. Therefore, the phenol VEG at ωB97MV/MM lies within the broad experimental spectra, while the phenolate VEG estimate at ωB97MV/MM is overestimated by 0.71 eV, which can be corrected by a constant shift. In this work, we did not use any correction factors or shifts.

Our best VEG estimate for indole at ωB97MV/MM is 7.79 eV, close to the experimental VIE of 7.4 ± 0.1 eV.^60^ To the best of our knowledge, the VIE of lumiflavin in solution has not been experimentally measured. Overall, the VEGs estimated at ωB97MV/MM level of theory agree well with the experimental estimates, and the error falls within the limits of our method of choice. The close agreement with experimental or high-level computed VIEs reinforces the credibility of our DFT/MM approach.

### ML Model Performance

Table 3 illustrates the MAE and RMSE heatmaps for all ML models utilized in this study across different systems. The table also shows the scores for the combined systems, collectively denoted as “All”, and the models trained with data from all systems are termed “general” models. The details of the optimized hyperparameters in the models are provided in the SI (Table S6). The accuracy scores are presented for models using features computed with both HF/MM and EMP/MM methods for QM cutoff 0.0 (Table 3), and corresponding heatmaps for QM cutoff 7.5 scheme are provided in Figures S2 and S3.

**Table 3:**
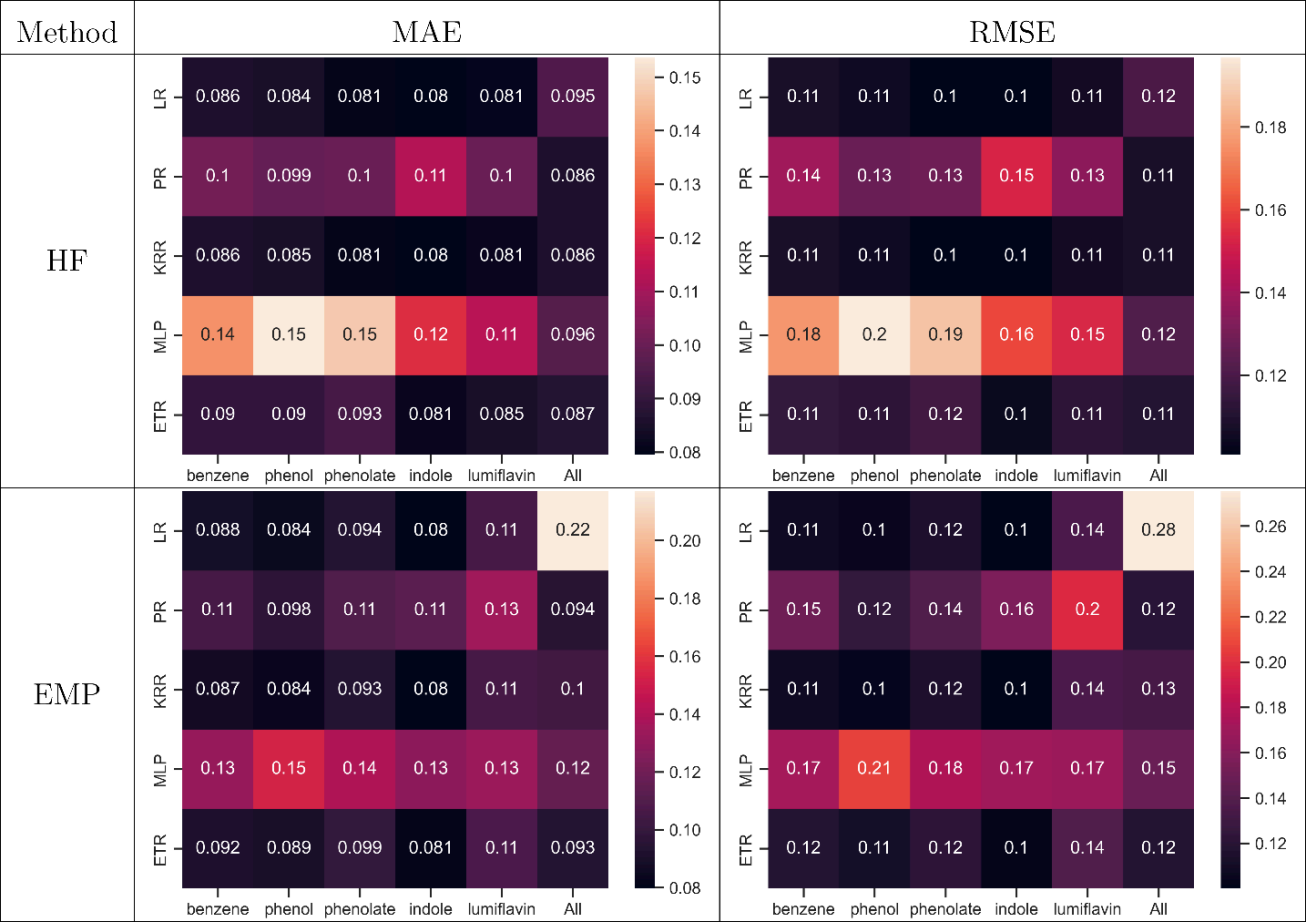
Scoring heatmaps for models trained on features from HF/MM and EMP/MM calculations with a QM cutoff 0.0. The RMSE and MAE are calculated using a five-fold cross validation scheme

Regardless of the QM method employed (single-point QM/MM) for feature calculation, models such as LR, KRR, and ETR consistently outperform PR and MLP for individual systems. In the case of general models (column labeled “All” in the heatmaps), ETR exhibits superior performance in both HF/MM and EMP/MM cases for both cutoff values, with MAE ranging from 0.08 to 0.09 eV for cutoff 0.0 (Table 3), and 0.04 to 0.07 eV for cutoff 7.5 (Figure S2). Consequently, the ETR model is selected as the optimal general model despite KRR showing marginally better MAE and RMSE values in HF/MM with a QM cutoff of 0.0 (Table 3).

Interestingly, while general LR performs reasonably well for HF/MM features, it emerges as the worst for EMP/MM with a cutoff of 0.0. Similarly, the LR model ranks as the worst general model for the 7.5 cutoff. This disparity might be due to the difference in the HF/MM and EMP/MM learning problems. HF/MM features simplify the learning problem for a linear model to capture the correlation between features and reference. In contrast, the EMP/MM features need a more sophisticated model to capture the correlation with DFT/MM level VEGs. The relatively good performance of LR for individual systems, with MAE ranging from 0.03–0.1 eV, can be explained by a similar rationale; individual learning tasks are inherently less “complex” than the comprehensive “All” system.

**Figure 3:**
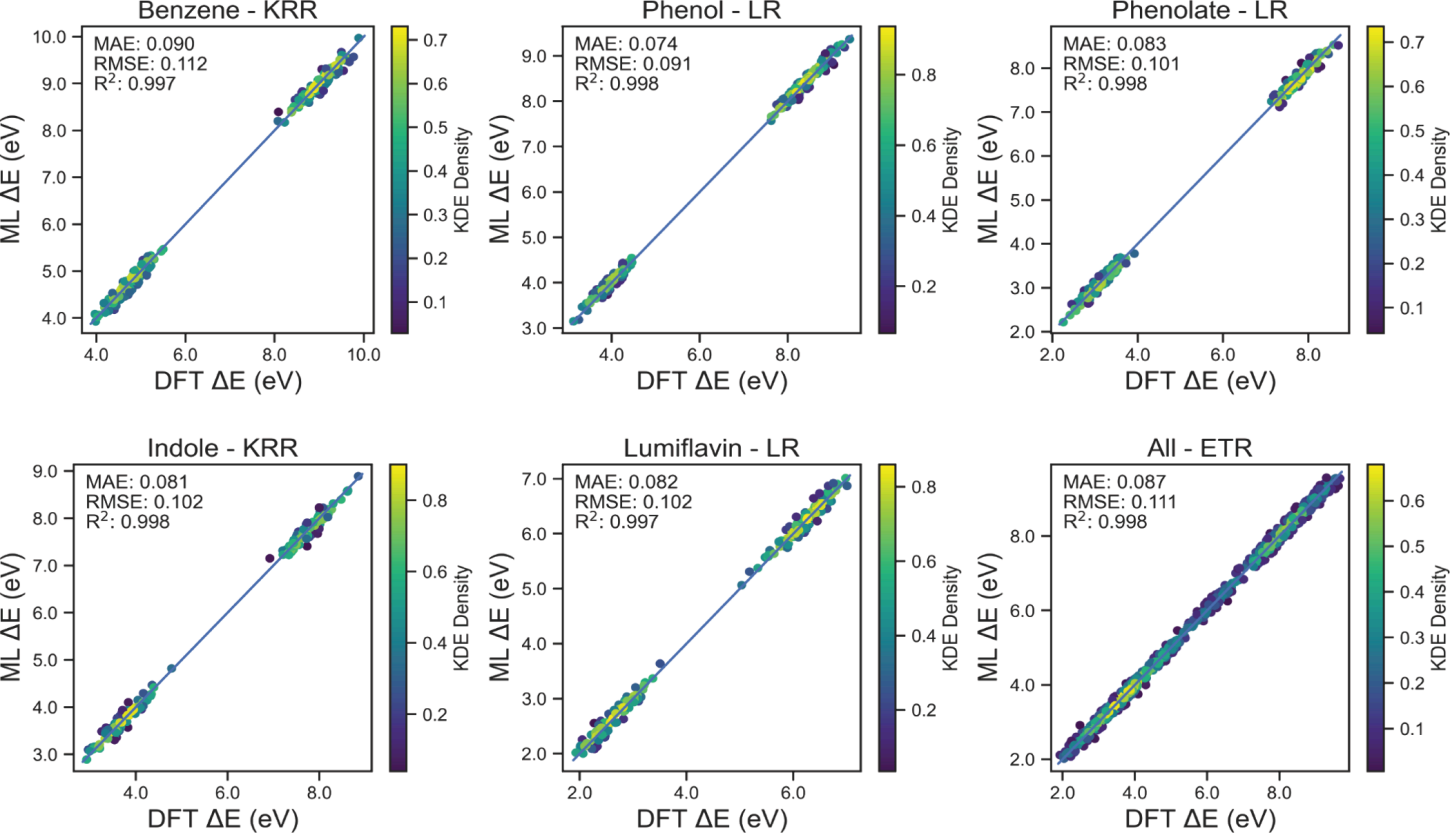
Parity plots for HF/MM with a QM cutoff 0.0 Å calculated for test set (20% of the data set) unseen by the model during training

Figure 3 shows the parity plots of the best model for each system for the HF/MM with QM cutoff 0.0. The parity plots for the EMP/MM cutoff 0.0 case, and the cutoff 7.5 plots are shown in the SI (Figures S4–S6). Each plot is a scatter plot of DFT/MM cutoff 7.5 VEGs and ML predictions for the test set (data set not involved in training the model), and each point is colored by its spatial density. The parity plots evaluate the out-of-sample predictive performance of the best model in each case. The best model in each case is selected using the scoring heatmap, i.e., the model that gives the lowest MAE and RMSE for an individual system is called its best model and for the general system, ETR is chosen as the best model. The figures also show the MAE, RMSE, and R^2^ scores for the predictions.

The difference in accuracy scores between the best models for HF/MM and EMP/MM methods is negligible, with MAE and RMSE around ∼0.1 eV. This MAE is close to what is achieved by other studies which use similar models and workflow as this work,^29,31,41,61,62^ for example, the study by Chen *et. al*(62) showed a MAE of 0.16 V for redox potential prediction of organic compounds from the ROP313 data set.^63^ This underscores the reliability of ML models trained using features extracted from the more cost-effective semiempirical HF-3c/MINIX method at 0.0 Å cutoff for predicting VEGs at the ωB97MV/6-31+G* level of theory with a larger QM cutoff radius in a QM/MM scheme. Notably, the HF/MM method at 7.5 Å QM cutoff, while being a less cost-effective method for feature extraction, presents as the most accurate model with MAE and RMSE around ∼0.05 eV.

We have also extended this approach on a smaller subset of our data set to test whether this strategy can be adopted for predicting VEGs at the post-HF level, such as the equation-of-motion ionization potential (EOM-IP-CCSD) method. Due to the high computational cost of EOM-IP-CCSD, we excluded lumiflavin and only included the reduced surface for the remaining systems in this analysis. Figure S7 shows the parity plot of the ETR model which shows an MAE of <0.05 eV in both HF/MM and EMP/MM cases. This demonstrates that features calculated at HF/MM or EMP/MM with a cutoff of 0.0 Å can predict EOM-IP-CCSD level VEGs at the same cutoff. Due to their very high computational expense, post-HF methods cannot be applied to larger QM cutoffs. In the next section, we investigate how the ML models used in this study depend on the training data size.

### Model Performance vs Training Data

The assessment of model performance with respect to training data is crucial for understanding the behavior of the ML models. Learning curves plot the cross-validated testing and training accuracy against the number of training examples, providing insights into the data dependence of the ML models. We generated the learning curves using the *learning_curve* function in the scikit-learn library. We only used the training set, i.e., 80% of our data set, generated earlier, and a 5-fold cross validation to plot the MAE score vs the number of training examples. The HF/MM and EMP/MM learning curves are shown in the SI for both cutoff 0.0 and 7.5 (Figures S8–S11).

We observe that, for individual models, the training and validation scores converge with a training set size of around 300–400 examples, but the curves show no signs of converging for the general model since they flatten after around 2000 training points. Both HF/MM and EMP/MM methods exhibit similar learning curves and similar dependence on number of training examples required to converge training and validation errors.

**Figure 4:**
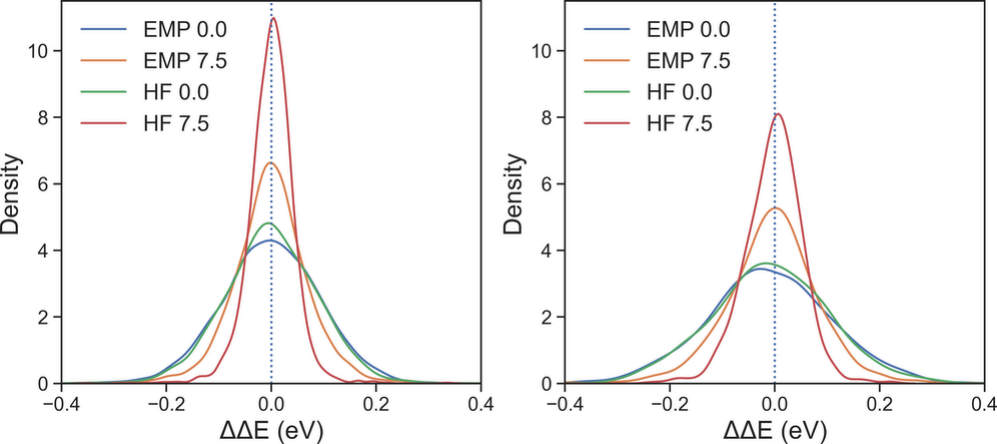
KDE plot of ΔΔE (=ΔE_*ML*_ - ΔE_*DFT*/*MM*_) for training (left) and validation (right) data. The legend mentions the method and QM cutoff value in Å.

The flattening of the training curve suggests that the ETR model has reached its MAE accuracy limit, which is approximately 0.06 eV, while the validation accuracy hovers around 0.09 eV. Such observations were attributed to the set of features and diversity of the data set for other chemical properties.^64,65^ In order to assess the overall performance of the general ETR models used in this work, we show the kernel density estimations (KDE) of the prediction errors, ΔΔE (ΔE_*ML*_ - ΔE_*DFT*_) for training and testing data sets in Figure 4. The ΔΔE KDEs for both train and test data sets are systematically distributed around 0 eV, with HF/MM with QM cutoff 7.5 showing the lowest deviation and EMP/MM with cutoff 7.5 showing the second best performance. Figure 4 also shows the training ΔΔE distribution has a smaller full width at half-maximum (fwhm) compared to the testing distribution, which is supported by the training curves. Interestingly, the difference between EMP/MM and HF/MM with a cutoff of 0.0 Å is negligible for both data sets, indicating no advantage in using HF over the EMP method for feature generation in case of 0.0 Å cutoff.

## Summary and Conclusions

In this article, we have explored ML methods to predict DFT/MM level VEGs for a set of biologically relevant molecules using features extracted from cheaper HF or EMP methods. We built multiple ML models for each system, ranging from linear regression and kernel-based methods to tree-based models, and calculated accuracy scores such as MAE and RMSE.

We observed that the differences in accuracy scores between models trained with features extracted from HF/MM with a QM cutoff of 0.0 and models that use features extracted from EMP/MM with a cutoff of 0.0 are negligible (<0.01 eV). This indicates that using the more expensive HF/MM calculation for feature extraction does not offer a significant advantage. We also calculate the prediction accuracy of models trained with features from HF/MM and EMP/MM with a cutoff of 7.5, which shows the best overall features are from HF/MM and 7.5 QM cutoff, with MAE ∼ 0.04 eV. However, HF/MM calculations at a QM cutoff of 7.5 are still costly, and the difference between the accuracy scores of EMP/MM with cutoff values of 7.5 and 0.0 is negligible. Therefore, the features extracted from the QM/MM single-point energy calculations at cutoff 0.0 are optimal for predicting accurate VEGs.

We also show that simple regression models, such as linear regression, show the best results for individual systems with an MAE of about 0.08–0.1 eV. In contrast, for the general system, tree-based models like ETR work the best with an MAE of about 0.1 eV. This observation generally holds regardless of the method used for the features. We also investigated the training data set size dependence using learning curves, which showed two different behaviors for individual and general systems. As the training set size increased, the training and validation curves converged for all individual systems. We observed that the ETR model shows a gap between the training and validation curves for the general system. However, we do not observe an increase in validation error for the general system, which may result from the model reaching its accuracy limit for our specific featurization method. This analysis is supported by the ETR learning curves of cutoff 7.5 models, where the gap between training and validation curves is smaller than the cutoff 0.0 models.

Overall, the accuracy scores of these models are on par with other ML models for predicting redox properties. Furthermore, our models are sensitive to the conformations of the solute and explicit solvents, providing a way to ensemble average an electronic property. Since the features are collected from a very cheap QM/MM calculation with a tiny QM region, the ML models are essentially correcting for (i) QM size in QM/MM calculation, (ii) spin contamination for open-shell species, and (iii) solvent orientation around solute at a much cheaper cost. Therefore, this approach can potentially allow extensive QM/MM sampling of electronic properties (VEG in this case), leading to a robust estimation of the observables.

## Data Availability

The code and relevant data can be downloaded from https://github.com/achary01su/ML-VEG.

## Supporting Information Available

Additional details of force field design for the nonstandard surface, QM/MM simulations, single-point calculations, feature selection and model details, model accuracy, prediction of VEGs at higher level of theory, and initial geometries of the molecules (PDF)
