# Supplementary material for: Machine Learning Approach to Vertical Energy Gap in Redox Processes
Source: J Chem Theory Comput. 2024 Jul 24;20(15):6747–55. doi: 10.1021/acs.jctc.4c00715 (PMC11325558; doi:10.1021/acs.jctc.4c00715)
Supplement: Supplementary file 1 — ct4c00715_si_001.pdf [file ct4c00715_si_001.pdf]

Supporting Information: Machine Learning Approach To Vertical Energy Gap in Redox Processes

Ronit Sarangi,[†] Suman Maity,[†] and Atanu Acharya[*,†,‡]

†*Department of Chemistry, Syracuse University, Syracuse, NY 13244, United States*

‡*BioInspired Syracuse, Syracuse University, Syracuse, NY 13244, United States*

E-mail: achary01@syr.edu

Contents

1 Force field for initial molecular dynamics **S-3**

2 Analysis of the QM/MM setup and calculations **S-3**

3 Feature selection and model details **S-7**

4 Model accuracy **S-8**

4.1 Cutoff 7.5 Å ML score heatmaps S-8

4.2 Cutoff 0.0 Å EMP/MM parity plot S-9

4.3 Cutoff 7.5 Å parity plots S-10

5 VEG predictions at higher level of theory **S-11**

6 Learning curves for each model **S-12**

6.1 Cutoff 0.0 Å learning curves S-12

6.2 Cutoff 7.5 Å learning curves S-13

7 Optimized geometry **S-14**

References **S-19**

1 Force field for initial molecular dynamics

The force field parameters were derived based on CHARMM general force field (CGenFF).[S1] We have taken the initial force field parameters for the reduced ground state (oxidized state for lumiflavin) from the CGenFF[S1] and modified the parameters for both oxidized and reduced states based on the optimized geometry of these molecules. Such an approach was used in prior studies of similar molecules.[S2,S3]

The geometries of the oxidized and reduced states were optimized in the gas phase. Geometry optimization and NBO calculations were performed at ωB97XD/6-31+G* level of theory using Gaussian 16.[S4] The equilibrium bond lengths and bond angles were obtained from the optimized geometry of the corresponding state. In the optimized geometry, we have calculated natural bond orbital (NBO) charges and used them as the partial charges in the modified force field parameters. The force constants and the rest of the nonbonded parameters were directly used from CGenFF, and they remained the same for both states.

2 Analysis of the QM/MM setup and calculations

Table S1: Average number of waters in the QM region for cutoff 7.5 single point calculations

System	Surface	Number of waters[a]
Benzene	Reduced	52.8 ± 3.0
	Oxidized	54.2 ± 2.8
Phenol	Reduced	53.3 ± 2.9
	Oxidized	54.4 ± 2.6
Phenolate	Reduced	55.5 ± 2.8
	Oxidized	53.1 ± 3.0
Indole	Reduced	52.5 ± 2.8
	Oxidized	54.0 ± 2.8
Lumiflavin	Reduced	47.8 ± 2.7
	Oxidized	48.8 ± 2.6

[a]: The average number of waters in the QM region and its standard deviation is calculated across 500 snapshots for each surface.

Table S2: Number of QM waters vs VEG for all systems with a QM cutoff of 7.5 Å. The "Frames" column indicates the number of snapshots with the respective number of waters in the QM region. ΔE statistics are provided for the top three water populations.

Surface	# waters	Frames	Average ΔE (eV)
Reduced Benzene	51	46	9.01 ± 0.34
	52	66	9.04 ± 0.32
	53	74	9.03 ± 0.32
Oxidized Benzene	53	71	4.73 ± 0.33
	54	69	4.76 ± 0.34
	55	63	4.78 ± 0.28
Reduced Phenol	53	86	8.51 ± 0.35
	54	60	8.44 ± 0.38
	55	60	8.49 ± 0.30
Oxidized Phenol	53	67	3.94 ± 0.31
	54	83	3.92 ± 0.31
	55	61	3.92 ± 0.28
Reduced Phenolate	53	68	7.77 ± 0.30
	54	78	7.77 ± 0.37
	55	70	7.79 ± 0.37
Oxidized Phenolate	52	69	3.14 ± 0.33
	53	76	3.12 ± 0.34
	54	61	3.10 ± 0.34
Reduced Indole	52	57	7.81 ± 0.38
	53	60	7.70 ± 0.38
	54	63	7.79 ± 0.30
Oxidized Indole	53	66	3.63 ± 0.36
	54	66	3.65 ± 0.31
	55	75	3.69 ± 0.33
Reduced Lumiflavin	53	71	6.22 ± 0.33
	54	69	6.25 ± 0.34
	55	63	6.23 ± 0.28
Oxidized Lumiflavin	46	66	2.60 ± 0.37
	47	73	2.55 ± 0.32
	48	79	2.62 ± 0.37

Table S3: Effect of number of QM waters on one representative snapshot (ΔE closest to the mean) for each system in its reduced surface.

Name	# QM waters	ΔE (eV)
	52	8.827
Benzene	54	8.846
	56	8.837
	52	7.467
Phenol	54	7.462
	56	7.463
	53	7.127
Phenolate	55	7.137
	57	7.121
	52	7.736
Indole	54	7.736
	56	7.720
	48	3.006
Lumiflavin	50	3.009
	52	3.012

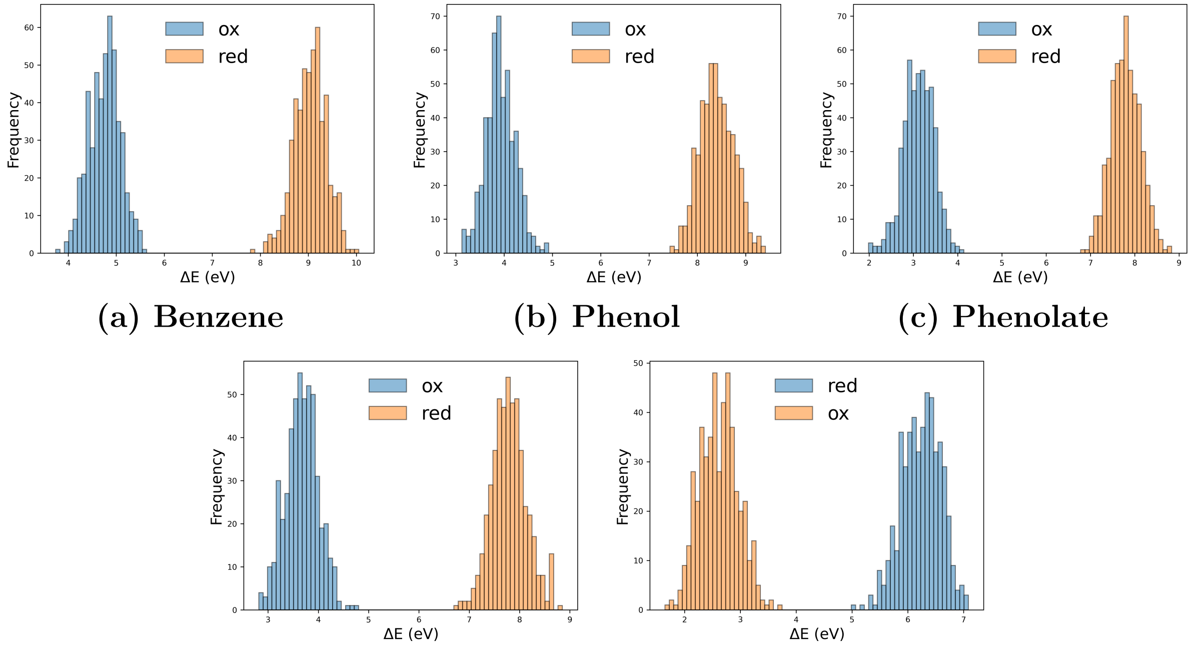

(a) Benzene **(b) Phenol** **(c) Phenolate**

(d) Indole **(e) Lumiflavin**

Figure S1: ΔE distribution at the reduced and oxidized surfaces from DFT/MM calculations with a 7.5 Å QM cutoff.

Table S4: EMP/MM VEGs (in eV) for all systems. We used the HF-3c/MINIX semiempirical method in this study.

System	Surface	cutoff 0.0	cutoff 7.5
Benzene	Reduced	8.45 ± 0.37	8.31 ± 0.37
	Oxidized	3.61 ± 0.37	3.51 ± 0.38
Phenol	Reduced	7.77 ± 0.45	7.40 ± 0.45
	Oxidized	2.69 ± 0.33	2.43 ± 0.33
Phenolate	Reduced	3.48 ± 0.40	4.21 ± 0.45
	Oxidized	-1.49 ± 0.37	-1.04 ± 0.42
Indole	Reduced	6.77 ± 0.42	6.49 ± 0.42
	Oxidized	1.98 ± 0.42	1.76 ± 0.42
Lumiflavin	Reduced	5.47 ± 0.46	5.76 ± 0.48
	Oxidized	1.33 ± 0.41	1.59 ± 0.43

Table S5: Average spin multiplicity ($\langle S^2 \rangle$) of all systems calculated using ωB97MV/MM, HF/MM and EMP/MM for "cutoff 0.0". The 6-31+G* basis set was used for DFT and HF QM calculations, while the MINIX (minimal) basis set was used for EMP (HF-3c) calculations.

		$\langle S^2 \rangle$ at cutoff 0.0		
Name	Surface	ωB97MV/MM	HF/MM	EMP/MM
Benzene	Reduced	0.77 ± 0.001	0.88 ± 0.016	0.90 ± 0.024
	Oxidized	0.77 ± 0.001	0.88 ± 0.022	0.90 ± 0.031
Phenol	Reduced	0.77 ± 0.002	1.03 ± 0.080	1.20 ± 0.096
	Oxidized	0.78 ± 0.002	1.09 ± 0.050	1.09 ± 0.050
Phenolate	Reduced	0.79 ± 0.004	1.32 ± 0.054	1.56 ± 0.052
	Oxidized	0.80 ± 0.004	1.34 ± 0.050	1.56 ± 0.052
Indole	Reduced	0.78 ± 0.003	1.25 ± 0.076	1.45 ± 0.100
	Oxidized	0.78 ± 0.004	1.32 ± 0.064	1.46 ± 0.079
Lumiflavin	Reduced	0.78 ± 0.034	1.43 ± 0.121	1.64 ± 0.148
	Oxidized	0.78 ± 0.005	1.40 ± 0.060	1.60 ± 0.074

3 Feature selection and model details

This section describes the details of the feature selection scheme used in this study.

- Nuclear repulsion energy, E_{NN}: It contains information about molecular size and structure, i.e., same molecule in different conformations will have different NREs,

$$E_{NN} = \frac{1}{2}\sum_{A}^{N}\sum_{B}^{N}\frac{Z_A Z_B}{R_{AB}}, \tag{1}$$

  where A and B are indices running over the solute molecule only, i.e., we do not take the solvent (water) into account when calculating NRE.

- HOMO energy (ϵ_{HOMO}): It is related to ionization energy through Koopmans' theorem and is extracted from Q-Chem output files in atomic units.

- LUMO energy (ϵ_{LUMO}): LUMO energy is also extracted from Q-Chem output file in atomic units.

- Dipole moments (μ): Dipole moment relays information about the polarity of the QM region. It is extracted from Q-Chem output file in Debyes.

- Charge-charge energy (E_{cc}): It measures the coulomb interaction energy of the MM region in the QM/MM single-point energy calculations. The formula is the same as NRE, but the indices run over all the MM atoms.

- Electrostatic potentials (ESP5 and ESP10): This quantity is calculated using the `PMEPot` plugin[S5] in VMD which outputs electrostatic potential in a grid for our system. We select the points within 5 Å and 10 Å of our solute and sum the potentials to obtain ESP5 and ESP10, respectively. These terms are supposed to represent the effect of the environment on the solute.

- Total SCF energy ($E_{tot} = E_{QM} + E_{QM-MM}$): The total SCF energy is obtained from the Q-Chem output file in atomic units.

Table S6: ML model names and hyperparameters

Model Name	Hyperparameters	Value
Linear Regression (LR)	-	-
Polynomial Regression (PR)	Degree	3
Kernel Ridge Regression (KRR)	alpha	1e-5
	gamma	1e-2
	kernel	rbf
Multilayer Perceptron (MLP)	hidden_layer_sizes	(80,60)
	activation	relu
	alpha	0.1
	epsilon	1e-5
Extra Trees Regressor (ETR)	max_features	0.8
	min_sample_split	8
	min_sample_leaf	3
	n_estimators	200

4 Model accuracy

4.1 Cutoff 7.5 Å ML score heatmaps

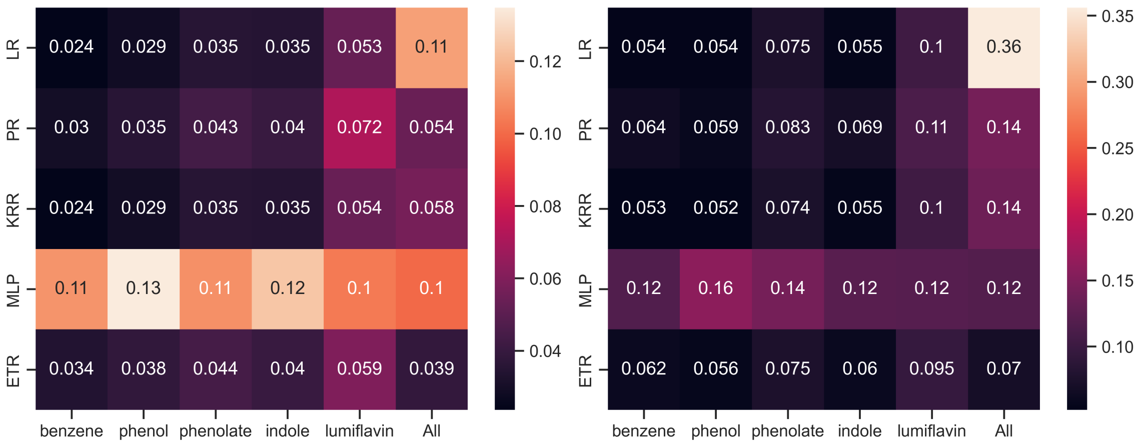

Figure S2: MAE heatmap for HF (left) and EMP (right)

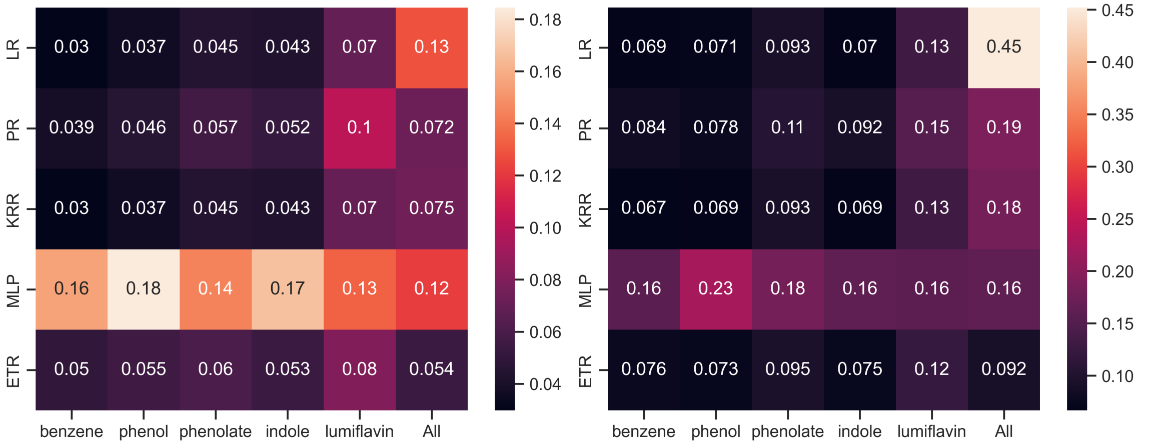

Figure S3: RMSE heatmap for HF (left) and EMP (right)

4.2 Cutoff 0.0 Å EMP/MM parity plot

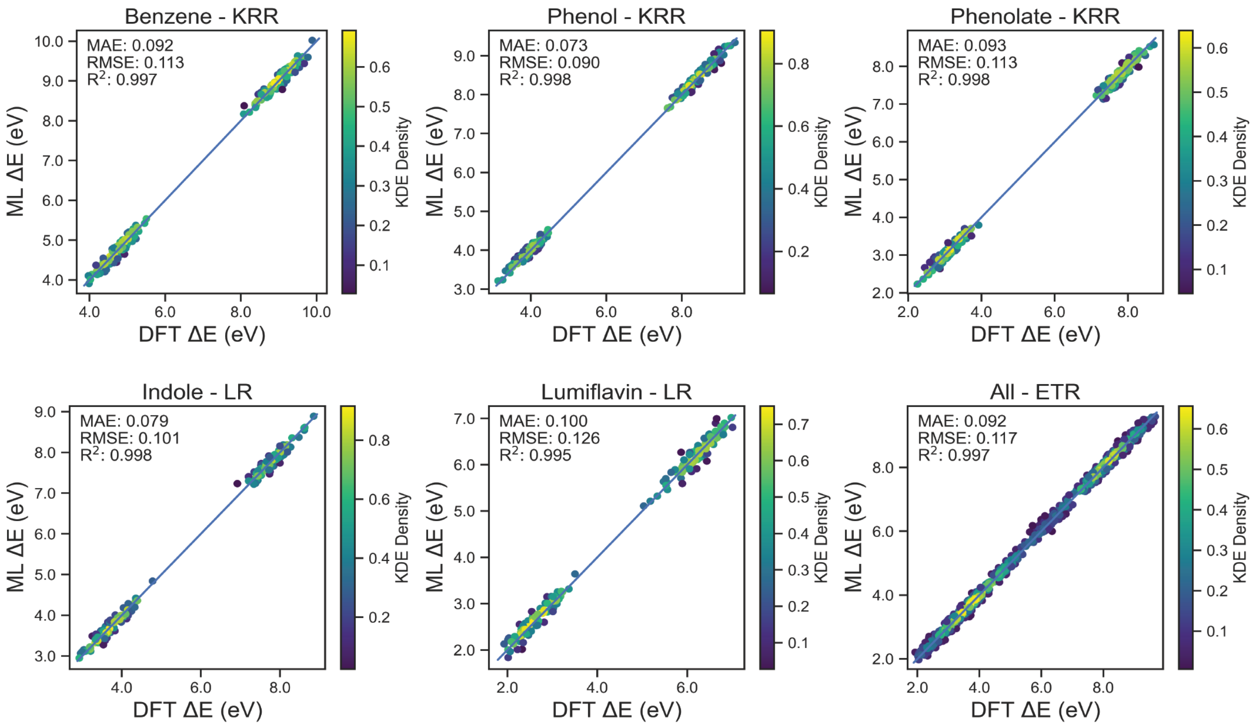

Figure S4: Parity plots for EMP/MM with a QM cutoff 0.0 Å calculated for test set (20% of the dataset) unseen by the model during training

4.3 Cutoff 7.5 Å parity plots

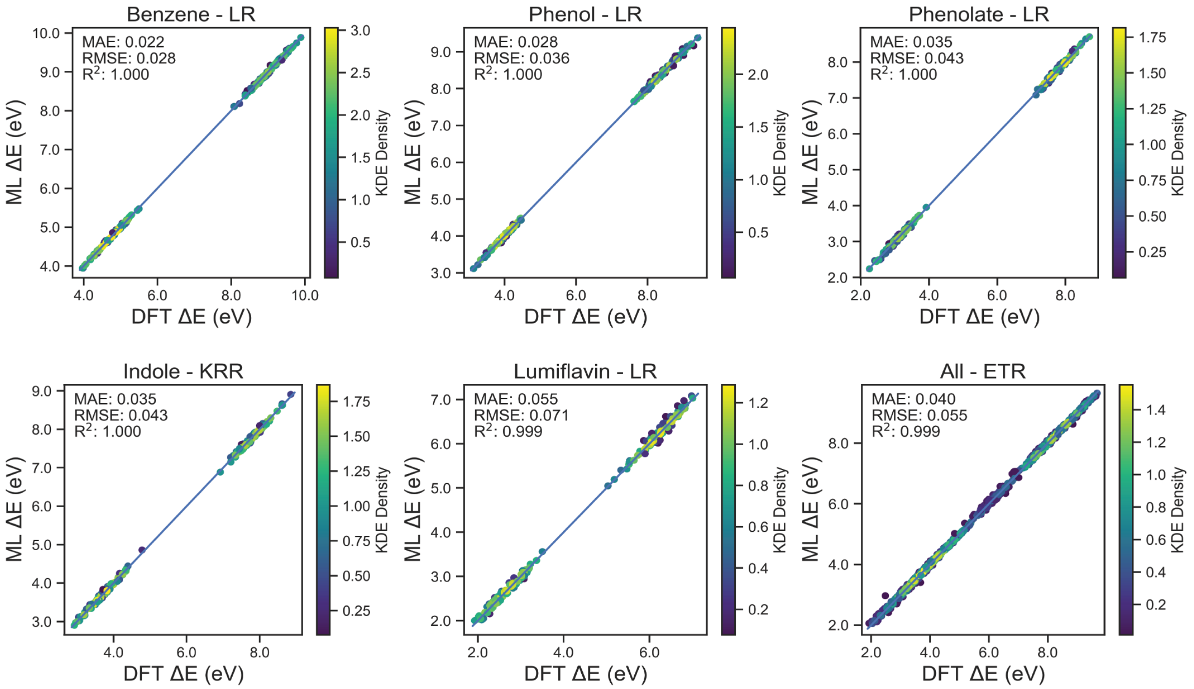

Figure S5: Parity plots for HF cutoff 7.5 Å calculated for test set not seen by the model during training.

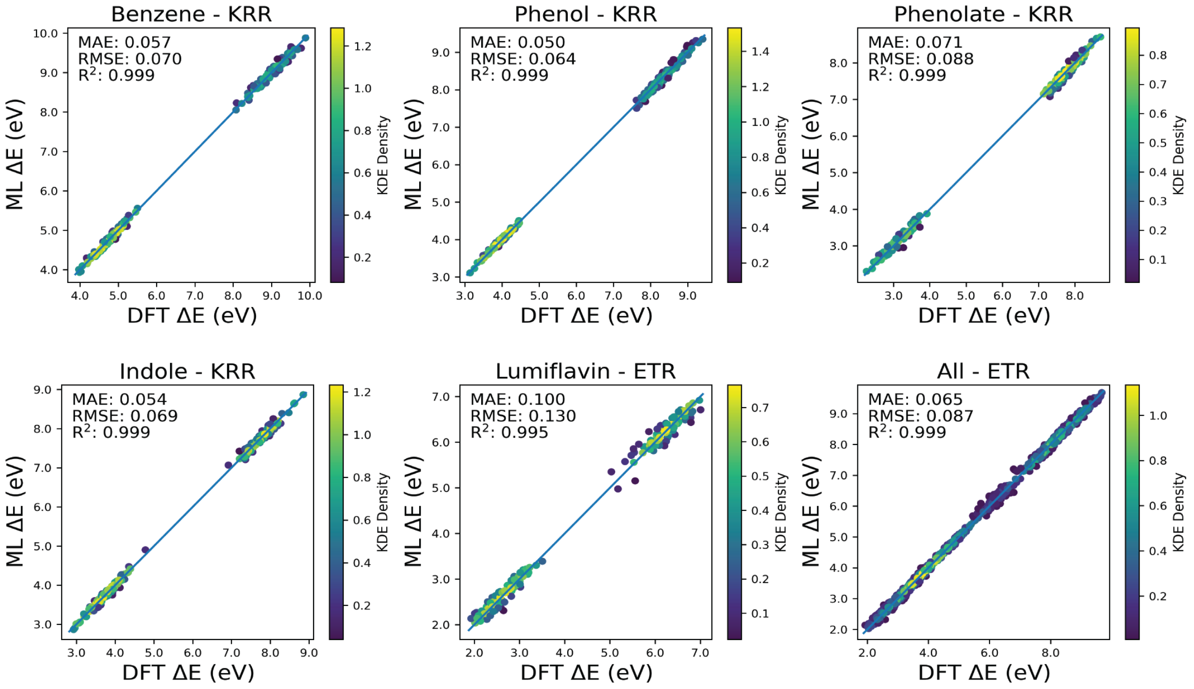

Figure S6: Parity plots for EMP cutoff 7.5 Å calculated for test set not seen by the model during training.

5 VEG predictions at higher level of theory

Here we take our current approach and apply it to predict post-HF level VEGs. Specifically, we build an ETR model to predict equation-of-motion ionization potential (EOM-IP) level VEGs ($\langle E_{ox} - E_{red} \rangle_{red}$) calculated at a QM cutoff of 0.0 Å, using HF and EMP features at the same cutoff. The cost of EOM-IP scales as $\mathcal{O}(N^6)$, where N is the number of basis functions. This high computational cost rules out the reduced lumiflavin, which has 385 basis functions (at QM cutoff 0.0 Å) with the 6-31+G* basis set. To minimize computational costs, we performed EOM-IP-CCSD calculations only for the reduced states of the rest of the systems, i.e., benzene, phenol, phenolate, and indole, and show the parity plot in Figure S7.

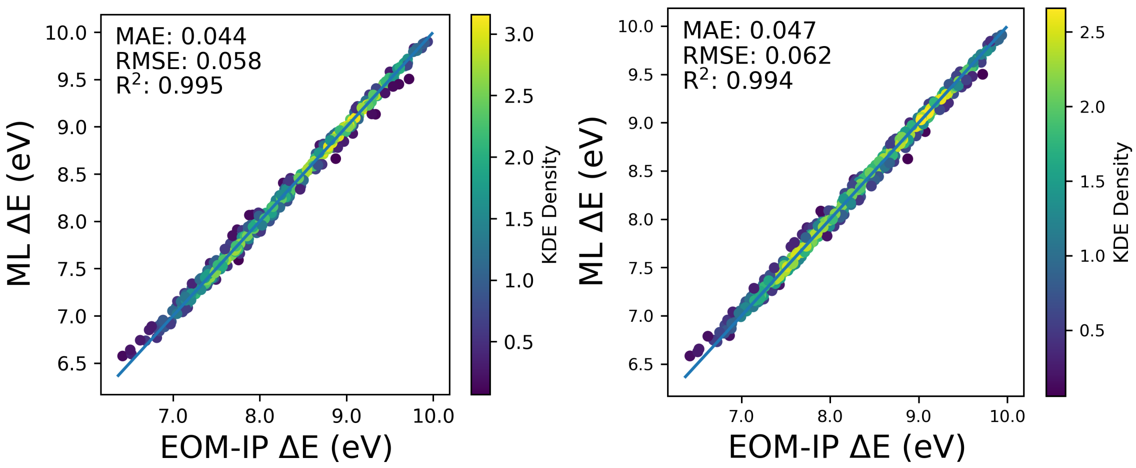

Figure S7: Parity plot for prediction of EOM-IP-CCSD level VEGs using HF (left) and EMP (right) features with ETR ML model. Both EOM-IP-CCSD calculations and the features use a QM cutoff 0.0 Å.

6 Learning curves for each model

6.1 Cutoff 0.0 Å learning curves

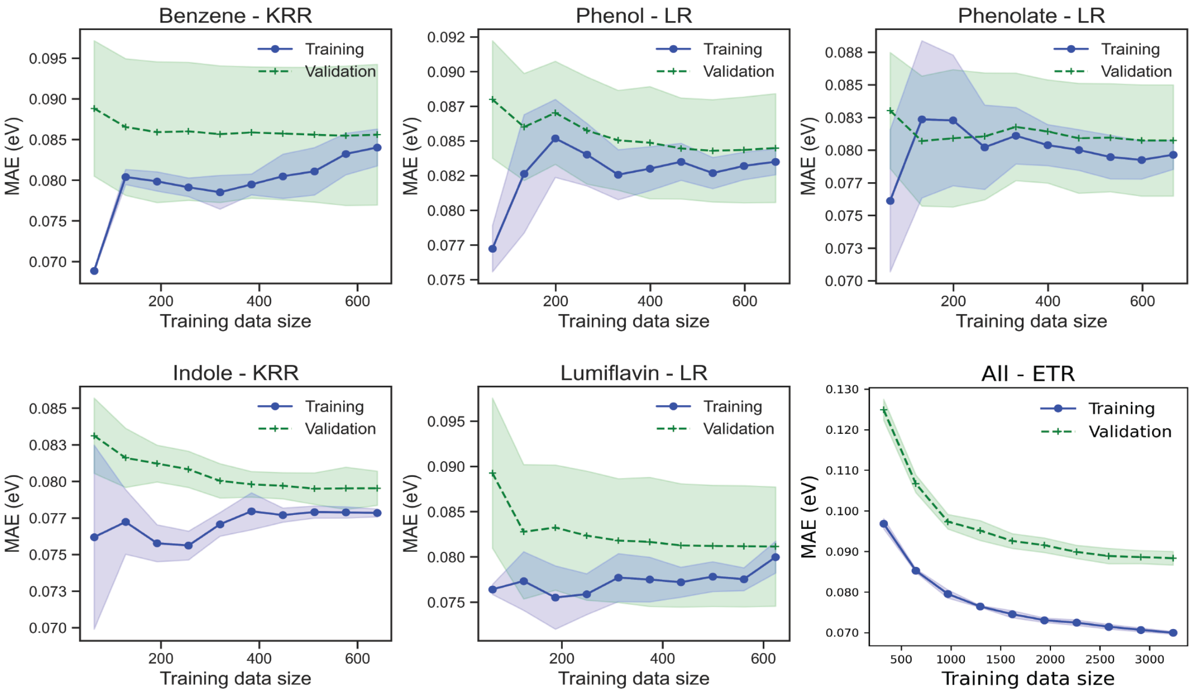

Figure S8: Learning curves for HF cutoff 0.0 Å using a 5 fold-cross validation scheme

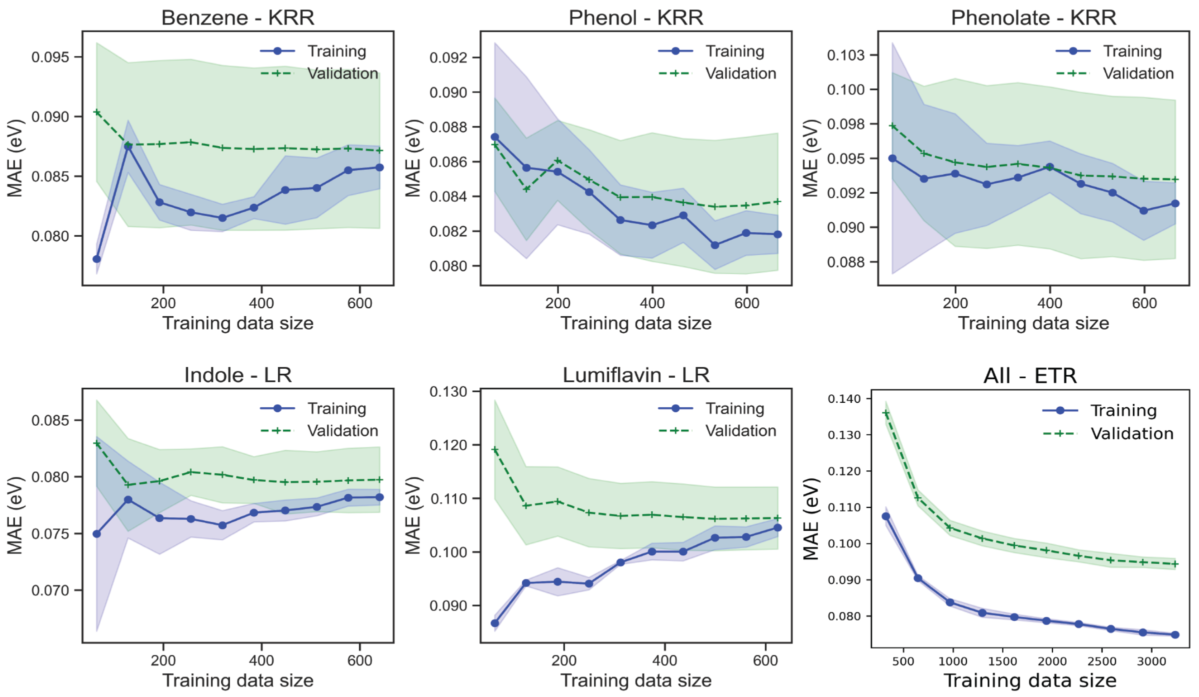

Figure S9: Learning curves for EMP cutoff 0.0 Å using a 5 fold-cross validation scheme

6.2 Cutoff 7.5 Å learning curves

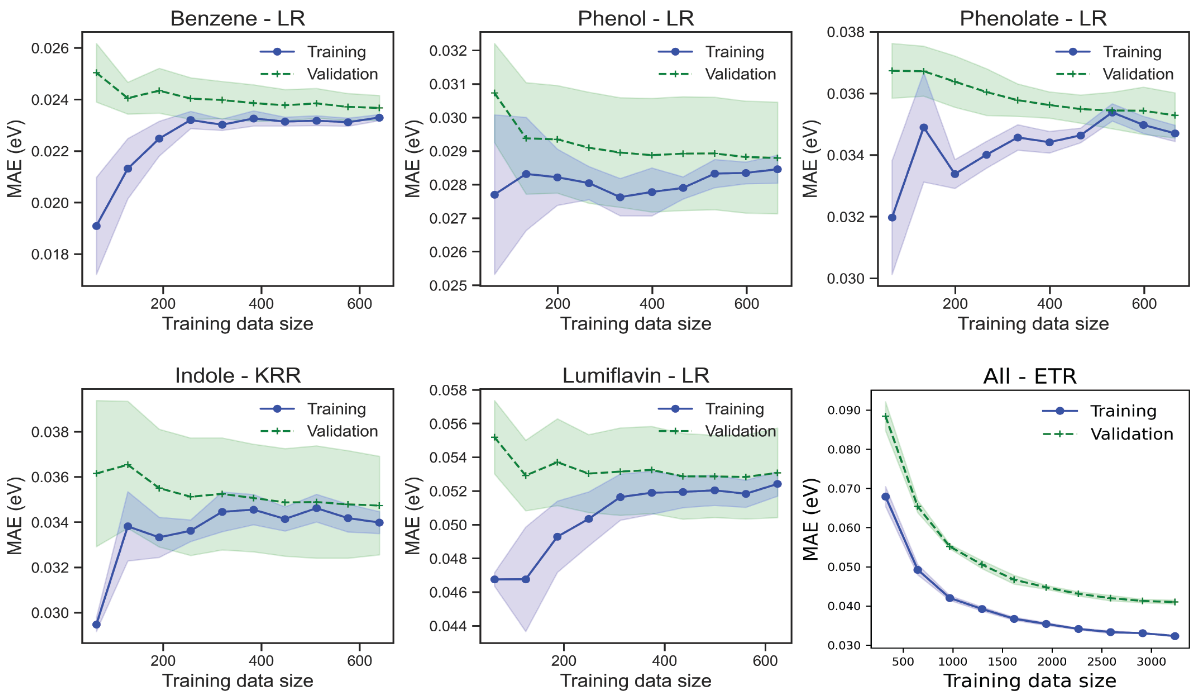

Figure S10: Learning curves for HF cutoff 7.5 Å using a 5 fold-cross validation scheme

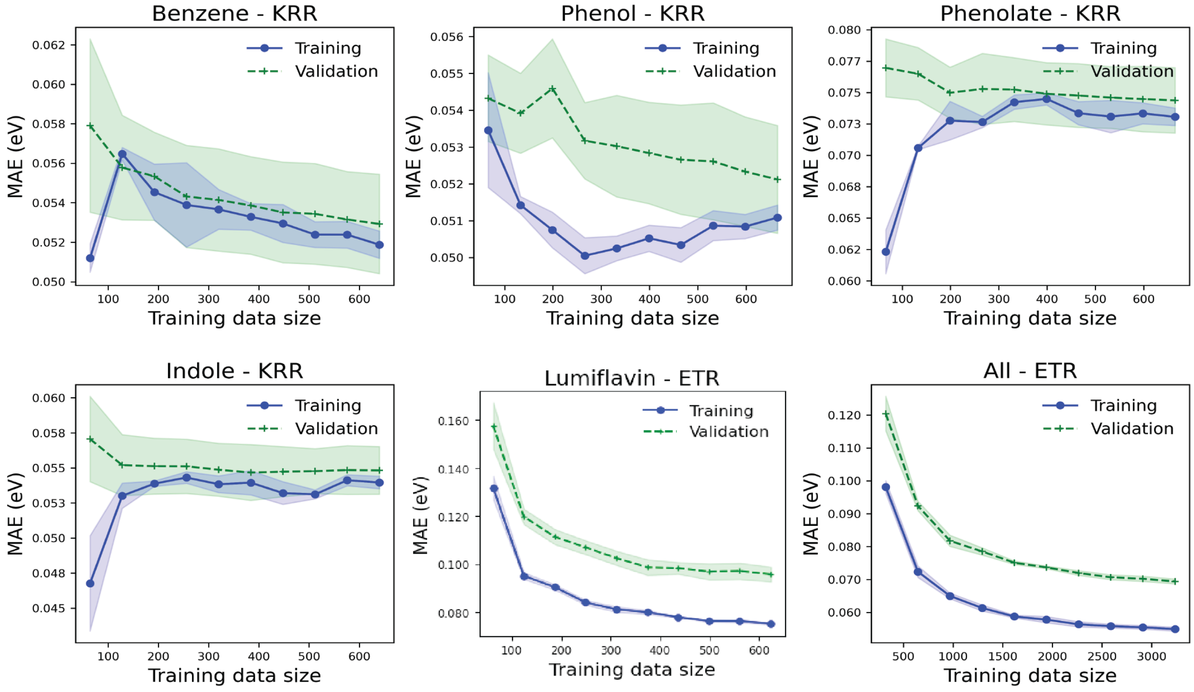

Figure S11: Learning curves for EMP cutoff 7.5 Å using a 5 fold-cross validation scheme

7 Optimized geometry

1. Benzene (reduced)

C	-1.321199	-0.445676	0.000001
C	-0.274574	-1.367017	-0.000008
C	1.046563	-0.921343	0.000013
C	1.321181	0.445730	-0.000001
C	0.274629	1.367005	-0.000010
C	-1.046600	0.921301	0.000007
H	-2.350707	-0.793030	-0.000000
H	-0.488628	-2.432248	-0.000018
H	1.862130	-1.639248	0.000003
H	2.350732	0.792957	-0.000003
H	0.488556	2.432263	-0.000004
H	-1.862080	1.639305	0.000014

2. Benzene cation (oxidized)

C	0.732273	-1.187315	-0.000006
C	1.436631	0.007648	-0.000004
C	0.718306	1.195190	0.000009
C	-0.732288	1.187314	-0.000006

C	-1.436631	-0.007645	-0.000004
C	-0.718291	-1.195191	0.000010
H	1.256909	-2.138200	-0.000010
H	2.520826	0.013971	-0.000014
H	1.232449	2.151819	0.000031
H	-1.256897	2.138213	-0.000015
H	-2.520826	-0.014003	-0.000008
H	-1.232460	-2.151806	0.000023

3. Phenolate (reduced)

C	-1.099284	-1.198964	-0.000052
C	0.288336	-1.210403	-0.000046
C	1.075784	-0.000016	0.000292
C	0.288329	1.210400	-0.000039
C	-1.099275	1.198977	-0.000062
C	-1.826984	-0.000001	0.000074
H	-1.636076	-2.149863	-0.000101
H	0.832913	-2.154120	-0.000151
H	0.832954	2.154089	-0.000137
H	-1.636080	2.149868	-0.000101
H	-2.915112	0.000010	0.000111
O	2.344996	0.000008	-0.000077

4. Phenolate radical (oxidized)

C	-1.084717	-1.224604	-0.000011
C	0.290397	-1.239886	-0.000011
C	1.046258	-0.000003	0.000069
C	0.290399	1.239881	-0.000050
C	-1.084712	1.224609	0.000020
C	-1.780790	0.000001	0.000007
H	-1.642326	-2.156710	-0.000032
H	0.856997	-2.165976	-0.000151
H	0.857008	2.165967	-0.000169
H	-1.642322	2.156715	0.000014
H	-2.866840	0.000005	0.000076
O	2.297059	0.000001	0.000015

5. Phenol (reduced)

C	1.153017	-1.205566	0.005527
C	-0.240449	-1.209319	-0.014843
C	-0.932133	0.000048	-0.013928
C	-0.240337	1.209372	-0.014768

C	1.153103	1.205513	0.005558
C	1.853084	-0.000069	0.018086
H	1.692383	-2.148676	0.006184
H	-0.800838	-2.139215	-0.038593
H	-0.800717	2.139274	-0.038402
H	1.692582	2.148557	0.006261
H	2.939002	-0.000095	0.031874
O	-2.314160	0.000218	-0.085645
H	-2.686840	-0.001458	0.804043

6. Phenol cation (oxidized)

C	1.103752	-1.251471	-0.000017
C	-0.263033	-1.245262	-0.000009
C	-0.940036	0.016091	-0.000005
C	-0.215716	1.255643	-0.000037
C	1.152179	1.221993	-0.000032
C	1.824192	-0.023944	0.000009
H	1.647206	-2.190266	-0.000002
H	-0.834688	-2.169296	-0.000035
H	-0.781649	2.181422	0.000048
H	1.724450	2.143207	0.000077
H	2.909524	-0.047063	0.000211
O	-2.240598	0.121963	0.000024
H	-2.708086	-0.732016	0.000063

7. Indole (reduced)

C	0.493366	0.613809	0.000000
C	-0.000000	-0.712452	0.000000
C	-1.368306	-1.002505	0.000000
C	-2.247292	0.068306	0.000000
C	-1.778974	1.398224	0.000000
C	-0.422870	1.678291	0.000000
C	1.927846	0.526255	0.000000
C	2.245060	-0.802083	0.000000
H	-1.730388	-2.027199	0.000000
H	-3.317084	-0.120092	0.000000
H	-2.497045	2.213256	0.000000
H	-0.070330	2.706247	0.000000
H	2.630755	1.347788	0.000000
H	3.211694	-1.286156	0.000000
N	1.089783	-1.554384	0.000000
H	1.050937	-2.560226	0.000000

8. Indole cation (oxidized)

C	0.486935	0.619189	0.000000
C	0.000000	-0.718521	0.000000
C	-1.334432	-1.024164	-0.000000
C	-2.235481	0.070546	-0.000000
C	-1.795761	1.397909	-0.000000
C	-0.438312	1.694317	-0.000000
C	1.884447	0.556976	0.000000
C	2.234452	-0.822679	0.000000
H	-1.704110	-2.044455	-0.000000
H	-3.300778	-0.136766	-0.000000
H	-2.524424	2.200783	-0.000000
H	-0.087979	2.721469	-0.000000
H	2.591506	1.375644	0.000000
H	3.217749	-1.274446	0.000000
N	1.127530	-1.558899	0.000000
H	1.104240	-2.571372	0.000000

9. Lumiflavin (oxidized)

N	-2.547472	1.303442	0.000348
C	-3.753278	0.633012	-0.000381
O	-4.827921	1.196869	-0.000980
N	-3.727972	-0.777448	-0.000524
C	-2.623993	-1.597520	0.000351
O	-2.698719	-2.807709	0.001257
C	-1.334661	-0.832391	0.000195
N	-0.225984	-1.492006	-0.000083
C	0.948238	-0.785697	-0.000216
C	2.161068	-1.497070	-0.000495
C	3.381027	-0.852591	-0.000387
C	4.668290	-1.635411	-0.000636
C	3.395809	0.567550	0.000022
C	4.708013	1.305791	0.000434
C	2.206299	1.282044	0.000250
C	0.967588	0.622698	0.000102
N	-0.239120	1.305113	0.000331
C	-1.433904	0.627878	0.000349
C	-0.247802	2.766443	0.000107
H	0.258758	3.138390	-0.894670
H	-1.284405	3.095469	-0.000714
H	0.257384	3.138818	0.895483
H	-4.634778	-1.230411	-0.000533
H	5.278111	-1.405592	0.881070
H	4.471359	-2.710629	-0.000171
H	5.277359	-1.406275	-0.883065

H	4.558860	2.388713	-0.001057
H	5.307728	1.045070	-0.879374
H	5.305610	1.047281	0.882395
H	2.249445	2.365174	0.000490
H	2.095375	-2.581403	-0.000746

10. Anionic lumiflavin (reduced)

N	-2.557982	1.299863	-0.000001
C	-3.757076	0.657706	-0.000008
O	-4.850390	1.230171	-0.000022
N	-3.734670	-0.740810	0.000000
C	-2.618214	-1.572491	0.000005
O	-2.764875	-2.794438	0.000015
C	-1.357932	-0.843253	0.000001
N	-0.197364	-1.545655	-0.000002
C	0.947710	-0.823579	-0.000001
C	2.189690	-1.496329	-0.000004
C	3.408206	-0.830889	-0.000004
C	4.702311	-1.608442	-0.000008
C	3.418319	0.576334	0.000000
C	4.718443	1.341470	-0.000003
C	2.200032	1.267360	0.000005
C	0.974411	0.600359	0.000004
N	-0.244532	1.280282	0.000010
C	-1.445037	0.573558	0.000003
C	-0.253742	2.727401	0.000011
H	0.255685	3.113769	-0.892490
H	-1.292226	3.053836	0.000016
H	0.255697	3.113767	0.892506
H	-4.634616	-1.202136	-0.000001
H	5.315013	-1.378312	0.882049
H	4.509576	-2.685945	-0.000011
H	5.315013	-1.378306	-0.882065
H	4.539345	2.422047	0.000031
H	5.331178	1.107357	-0.881542
H	5.331214	1.107306	0.881497
H	2.226486	2.352768	0.000011
H	2.148863	-2.583011	-0.000006

References

(S1) Vanommeslaeghe, K.; Hatcher, E.; Acharya, C.; Kundu, S.; Zhong, S.; Shim, J.; Darian, E.; Guvench, O.; Lopes, P.; Vorobyov, I.; others CHARMM general force field: A force field for drug-like molecules compatible with the CHARMM all-atom additive biological force fields. *J. Comput. Chem.* **2010**, *31*, 671–690.

(S2) Bogdanov, A. M.; Acharya, A.; Titelmayer, A. V.; Mamontova, A. V.; Bravaya, K. B.; Kolomeisky, A. B.; Lukyanov, K. A.; Krylov, A. I. Turning On and Off Photoinduced Electron Transfer in Fluorescent Proteins by π-Stacking, Halide Binding, and Tyr145 Mutations. *J. Am. Chem. Soc.* **2016**, *138*, 4807–4817.

(S3) Tazhigulov, R. N.; Bravaya, K. B. Free Energies of Redox Half-Reactions from First-Principles Calculations. *J. Phys. Chem. Lett.* **2016**, *7*, 2490–2495.

(S4) Frisch, M. J.; Trucks, G. W.; Schlegel, H. B.; Scuseria, G. E.; Robb, M. A.; Cheeseman, J. R.; Scalmani, G.; Barone, V.; Petersson, G. A.; Nakatsuji, H.; Li, X.; Caricato, M.; Marenich, A. V.; Bloino, J.; Janesko, B. G.; Gomperts, R.; Mennucci, B.; Hratchian, H. P.; Ortiz, J. V.; Izmaylov, A. F.; Sonnenberg, J. L.; Williams-Young, D.; Ding, F.; Lipparini, F.; Egidi, F.; Goings, J.; Peng, B.; Petrone, A.; Henderson, T.; Ranasinghe, D.; Zakrzewski, V. G.; Gao, J.; Rega, N.; Zheng, G.; Liang, W.; Hada, M.; Ehara, M.; Toyota, K.; Fukuda, R.; Hasegawa, J.; Ishida, M.; Nakajima, T.; Honda, Y.; Kitao, O.; Nakai, H.; Vreven, T.; Throssell, K.; Montgomery, J. A., Jr.; Peralta, J. E.; Ogliaro, F.; Bearpark, M. J.; Heyd, J. J.; Brothers, E. N.; Kudin, K. N.; Staroverov, V. N.; Keith, T. A.; Kobayashi, R.; Normand, J.; Raghavachari, K.; Rendell, A. P.; Burant, J. C.; Iyengar, S. S.; Tomasi, J.; Cossi, M.; Millam, J. M.; Klene, M.; Adamo, C.; Cammi, R.; Ochterski, J. W.; Martin, R. L.; Morokuma, K.; Farkas, O.; Foresman, J. B.; Fox, D. J. Gaussian˜16 Revision C.01. 2016; Gaussian Inc. Wallingford CT.

(S5) Aksimentiev, A.; Schulten, K. Imaging α-Hemolysin with Molecular Dynamics: Ionic Conductance, Osmotic Permeability, and the Electrostatic Potential Map. *Biophys. J.* **2005**, *88*, 3745–3761.
